# Identification of Novel Molecular Markers for Prognosis Estimation of Acute Myeloid Leukemia: Over-Expression of PDCD7, FIS1 and Ang2 May Indicate Poor Prognosis in Pretreatment Patients with Acute Myeloid Leukemia

**DOI:** 10.1371/journal.pone.0084150

**Published:** 2014-01-08

**Authors:** Yiming Tian, Zoufang Huang, Zhixiang Wang, Changxin Yin, Lanlan Zhou, Lingxiu Zhang, Kaikai Huang, Hongsheng Zhou, Xuejie Jiang, Jinming Li, Libin Liao, Mo Yang, Fanyi Meng

**Affiliations:** 1 Hematology Department of Nanfang Hospital, Southern Medical University, Guangzhou, Guangdong Province, China; 2 Bioinformatics Department, Southern Medical University, Guangzhou, Guangdong Province, China; 3 LKS Faculty of Medicine, The University of Hong Kong, Hong Kong, China; Albert Einstein College of Medicine, United States of America

## Abstract

Numerous factors impact on the prognosis of acute myeloid leukemia (AML), among which molecular genetic abnormalities are developed increasingly, however, accurate prediction for newly diagnosed AML patients remains unsatisfied. For further improving the prognosis evaluation system, we investigated the transcripts levels of PDCD7, FIS1, FAM3A, CA6, APP, KLRF1, ATCAY, GGT5 and Ang2 in 97 AML patients and 30 non-malignant controls, and validated using the published microarray data from 225 cytogenetically normal AML (CN-AML) patients treated according to the German AMLCG-1999 protocol. Real-time quantitative polymerase chain reaction and western blot were carried out, and clinical data were collected and analyzed. High Ang2 and FIS1 expression discriminated the CR rate of AML patients (62.5% versus 82.9% for Ang2, P = 0.011; 61.4% versus 82.2% for FIS1, P = 0.029). In CN-AML, patients with high FIS1 expression were more likely to be resistant to two courses of induction (P = 0.035). Overall survival (OS) and relapse-free survival (RFS) were shorter in CN-AML patients with high PDCD7 expression (P<0.001; P = 0.006), and PDCD7 was revealed to be an independent risk factor for OS in CN-AML (P = 0.004). In the analysis of published data from 225 CN-AML patients, PDCD7 remained independently predicting OS in CN-AML (P = 0.039). As a conclusion, Ang2 and FIS1 seem related to decreased CR rate of AML patients, and PDCD7 is associated with shorter OS and RFS in CN-AML. Hence, PDCD7, Ang2 and FIS1 may indicate a more aggressive form and poor prognosis of AML.

## Introduction

Acute myeloid leukemia (AML) is a clonal hematopoietic stem cell malignancy with highly heterogeneity. The prognostic prediction for AML had been improved tremendously in the past decades, however, accurate risk-stratification at diagnosis remained difficult. Meanwhile, molecular mechanisms in the etiology and progression of AML are still vague, and AML genomes is expected to lead to identification of even more prognostic markers [1].

Twenty-two molecular targets, which are potentially associated with complete remission (CR) durations of AML, has been selected based on our previous gene-chip study [2]. The study included 6 AML patients, three of them relapsed in 6–12 months and died within 3 months after relapse, the other three achieved and sustained CR status over 1 year. From the 22 targets, eight genes with clear gene fuctions were selected as prognostic candidates and investigated in our present study, including GGT5, FAM3A, PDCD7, FIS1, CA6, ATCAY, KLRF1 and APP.

Angiogenesis family play an important role in the growth and viability of myelogenous malignancies, and emerging data suggest a crucial involvement of the family member Angiopoietin-2 (Ang2) in this process. However, the role Ang2 played in AML remained controversial, such as the studies of Hou [3] and Loges [4] at mRNA level, and the reports both from Schliemann [5], [6] at protein level by immunohistochemistry and enzyme-linked immunosorbent assay (ELISA) respectively. With respects to this point, Ang2 was selected as another potential risk factor for AML in our study.

This study is aimed to investigate the expression status of the nine designated molecular markers including GGT5, FAM3A, PDCD7, FIS1, CA6, ATCAY, KLRF1, APP and Ang2, and their clinical relevance and prognostic significance in AML.

## Materials and Methods

### 2.1. Ethics statement

Human samples left over from clinical examinations were collected, and each patients were informed consent in written form for their samples to be stored and used for research purposes in advance, we didn't take any samples from patients specifically for this study. All patients data were collected and analyzed anonymously. This study was retrospectively authorized by Nanfang Hospital Ethics Committee (2012-178).

### 2.2. Patients and samples

Diagnostic bone marrow mononuclear cells were analyzed from 97 AML patients (age: 35 (13–65) years) with *de novo* (n = 93) or secondary AML (n = 4). All patients were diagnosed between September 2005 and July 2010 in *Nanfang Hospital*, Guangzhou, China. Diagnosis were based on the French-American-British (FAB) classification and World Health Orgnization (WHO) criteria [7], and the cutoff for blast count was 20%. Enrolled AML patients received standard combinations of anthracycline and cytarabine, subsequent induction was adopted until CR or allogeneic hematopoietic stem cell transplantation (allo-HSCT), and those acquired CR were consolidated optimally by regimens from induction or medium-/high- dose cytarabine (MDAC/HDAC). Eligible cases accepted allo-HSCT or autologous HSCT (auto-HSCT). CR patients were routinely given intrathecal injection of cytarabine or methotrexate (MTX) for 3–6 times. Follow-up of the patients were updated on 20^th^ May 2013. The clinical features of AML patients are listed in Table 1.

**Table 1:** Clinical features and treatment of 97 AML patients.

Gender, no.	
Male	54
Female	43
Age, Median (Range), years.	35 (13–65)
FAB subtype, no.	
M0	2
M1	8
M2	33
M4	19
M5	35
Cytogenetic risk group, no.	
Favorable	17
Intermediate	70
CN-AML	59
Adverse	10
Immunophenotyping, no.(%).	
CD34+	78 (83.9)
CD33+ or CD13+	92 (94.8)
CD14+ or CD11b+	29 (31.2)
CD117+	63 (67.7)
CD56+	22 (23.7)
Type of AML, no.	
De novo	93
Secondary	4
PB blasts, Median (Range), %	50 (0–99)
BM blasts, Median (Range), %	66 (23–98)
WBC count, Median (Range), G/l	45.2 (0.3–423)
LDH level, Median (Range), U/l	708 (93–3000)
Induction, no.	
DA	49
IA	28
TA	18
Death before induction	2
CR following induction therapy, no. (%).	64 (71.9)
Consolidation, no.	
Low intensity^a^	34
High intensity^b^	46
Death before CR or lost before consolidation	17
HSCT, no.	
Allo-HSCT	15
Auto-HSCT	7

AML acute myeloid leukemia, FAB French-American-British classification of acute myeloid leukemia, CN-AML cytogenetically normal acute myeloid leukemia, PB peripheral blood, BM bone marrow, CR, complete remission, DA daunorubicin and cytarabine, IA idarubicin and cytarabine, TA pirarubicin and cytarabine, MDAC/HDAC medium/high dose cytarabine, HSCT hematopoietic stem cell transplantation, Allo- allogeneic, Auto- autologous.

^a^ Patients received standard regimens from induction or <4 courses of MDAC/HDAC.

^b^ Patients received ≥4 courses of MDAC/HDAC or HSCT.

Bone marrow samples from 16 patients with iron deficiency anemia (IDA) and 14 patients with idiopathic thrombocytopenic purpura (ITP) were collected as control.

### 2.3. RNA extraction and reverse transcription

Total cellular RNA was extracted using Ficoll-Hypaque gradient (TBD, Tianjin, China) and reversely transcribed to cDNA by PrimeScript™RT Reagent Kit (TAKARA, Japan).

### 2.4. RQ-PCR

RQ-PCR was performed using SYBR Premix Ex Taq™(TaKaRa,Japan) according to the manufacturer's protocol on Mx3005P real-time PCR amplifier (Stratagene, USA). Relative mRNA amount was calculated as ***2^−ΔCt^ (ΔCt = Ct_target gene_ -Ct_β-actin_)*** [8].

### 2.5. Western blot

Western blot were carried out following the procedure described in our previous study [9], and primary antibodies included 1∶500 FIS1 rabbit monoclonal antibody (Sino Biological Inc, Beijing, China) and 1∶2000 β-actin mouse monoclonal antibody (Santa Cruz Biotechnology, Santa Cruz, CA).

### 2.6. Data mining from previously published microarrays

Data from 225 cytogenetically normal AML (CN-AML) patients treated according to the German AMLCG 1999 trial has been published on Gene Expression Omnibus (GEO, accession number, GSE12417). Datasets were mined for PDCD7 expression using Genebank accession number (AW953770) as published by National Center for Biotechnology Information (NCBI).

### 2.7. Data analysis

We assigned AML patients into high and low expression groups by median relative mRNA transcript levels for each gene. Primary refractory AML patients included those resistant to two courses of induction [10]. The definition of CR, Overall survival (OS) and Relapse-free survival (RFS) follow Cheson's criteria [11]. Kaplan–Meiyer curves and log-rank tests were used to estimate OS and RFS, a Cox proportional hazards model was constructed for OS in AML. Chi-square tests, Mann-Whiteney U tests and Spearman rank analysis were also performed. Two-tailed P<0.05 was considered as significant in primary analysis. Statistical analysis were performed with the statistical package SPSS 20.0 (Chicago, IL, USA).

For avoiding the interference from different cytogenetics, only CN-AML patients were included in survival analysis. CN-AML patients were divided into 3 groups by quartile of expression levels of target genes, and each patient was described by consolidation intensity. High intensity treatment included patients received ≥4 courses of MDAC/HDAC or HSCT, and low intensity included those received standard regimens from induction or <4 courses of MDAC/HDAC in consolidation (Table 1).

## Results

Expression data of PDCD7, FIS1, FAM3A, CA6, APP, KLRF1, ATCAY, GGT5 and Ang2 were analyzed in 97 AML patients and 30 non-malignant controls, as well as published data of PDCD7 expression from 225 CN-AML patients treated with a uniform protocol.

### 3.1. Association of expression levels of target genes with clinical features

**Figure 1:**
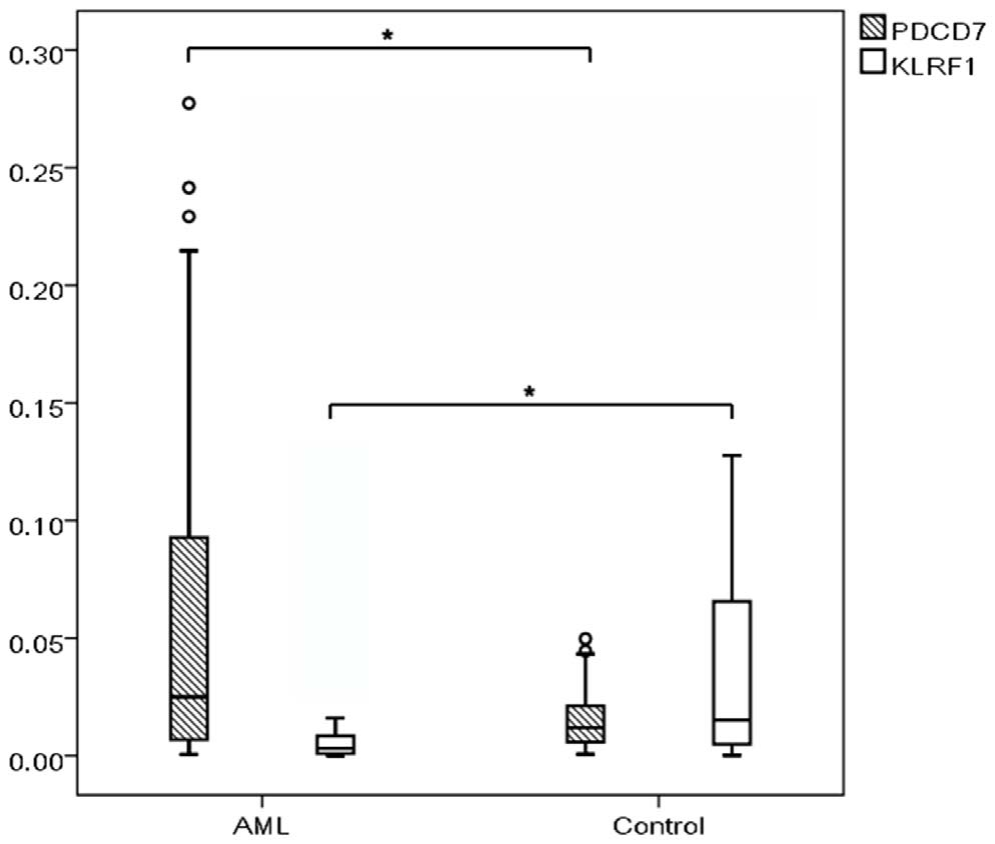
PDCD7 and KLRF1 expression range in AML (n = 97) and non-malignant controls (n = 30). *P<0.05.

**Figure 2:**
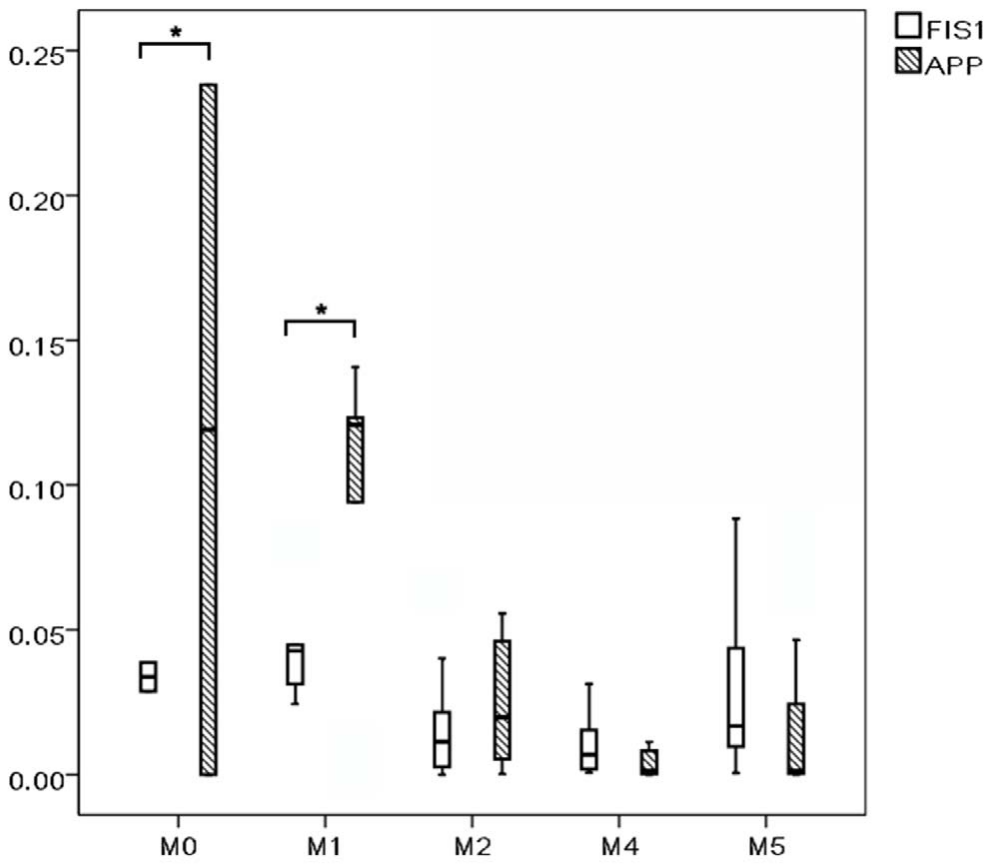
FIS1 and APP expression in different FAB subtypes. *P<0.05.

AML patients had higher PDCD7 and lower KLRF1 expression levels than those of non-malignant controls (P = 0.047, P = 0.001, Fig. 1), and patients with high FIS1 and APP expression were more likely to be classified as FAB M0/M1 subtype (P = 0.038, P = 0.018, Fig. 2), however, other targets had no similar results (P>0.05). PDCD7 expression levels positively correlated to bone marrow blast counts (r = 0.355, P<0.001). Expression levels of other target genes showed no correlations to age, gender, WBC, LDH levels, leukemic blasts in peripheral blood and bone marrow, immunophenotyping, leukemic infiltrations and cytogenetics (r<0.2, P>0.05).

### 3.2. Clinical outcome in 97 AML patients with respect to expression levels of target genes

**Figure 3:**
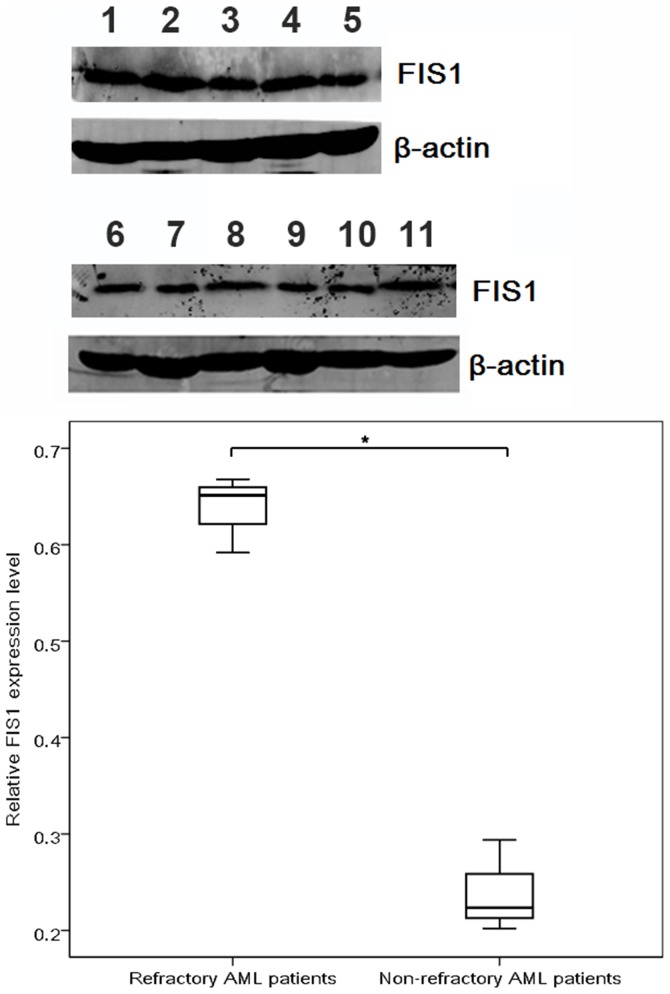
Immunobloting of FIS1 in primary refractory AML patients (1–5) and non-refractory AML patients (6–11). *P<0.05.

CR rate was elevated in patients with low Ang2 and FIS1 expression as compared to those with high expression levels, respectively (62.5% versus 82.9% for high versus low Ang2, P = 0.039; 61.4% versus 82.2% for high versus low FIS1, P = 0.029). Primary refractory AML patients tended to express higher FIS1 transcripts than non-refractory patients (P = 0.059), for CN-AML, the tendency became statistically significant (P = 0.035), and was validated at protein level (P = 0.001, Fig. 3). However, OS and RFS were not influenced by high versus low Ang2 and FIS1 expression in CN-AML (Ang2, OS: hazard ratio (HR) 1.046; 95% confidence interval (CI), 0.657–1.665; P = 0.851; RFS: HR, 1.471; 95% CI, 0.714–3.029; P = 0.295. FIS1, OS: hazard ratio (HR) 1.271; 95% confidence interval (CI), 0.812–1.988; P = 0.294; RFS: HR, 1.243; 95% CI, 0.665–2.323; P = 0.496).

**Figure 4:**
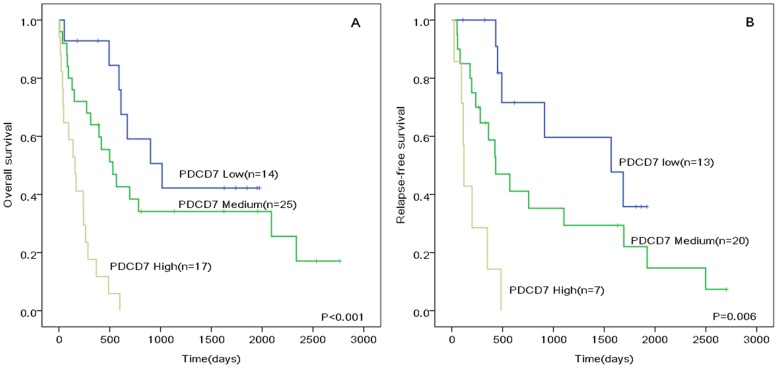
(A) Overall and (B) Relapse-free survival of analyzed patients with CN-AML according to different PDCD7 expression levels.

Patients with high PDCD7 expression had shorter OS and RFS in CN-AML, respectively (OS: hazard ratio (HR) 2.564; 95% confidence interval (CI), 1.628–4.039; P<0.001; RFS: HR, 3.343; 95% CI, 1.412–7.916; P = 0.006; Fig. 4). In multivariate analysis, when considering age (above or below 50 years), remission status (CR versus PR or NR) after two courses of induction and consolidation treatment (high versus low intensity), PDCD7 expression levels remained independently predicting OS for CN-AML (HR, 2.374; 95% CI, 1.317–4.277; P = 0.004, Table 2). However, CR rate seemed not influenced by PDCD7 expression (69.8% versus 73.9%, P = 0.664).

**Table 2:** Univariate and multivariate analysis for OS in 59 CN-AML patients.

Variables in the model	Univariate analysis			Multivariate analysis		
	HR	95% CI	P	HR	95% CI	P
Age above 50 versus ≤50 years	2.538	1.111–5.797	0.027	0.301	0.032–2.813	0.292
CR versus PR/NR after two inductions	0.529	0.278–1.044	0.067	0.636	0.284–1.422	0.27
Treatment high versus low intensity*	0.306	0.131–0.714	0.006	0.208	0.074–0.588	0.003
PDCD7 high versus medium versus low expression	2.564	1.628–4.039	<0.001	2.374	1.317–4.277	0.004

CN-AML cytogenetically normal acute myeloid leukemia, HR hazard ratio, CI coefficient index, CR complete remission, P P values, PR partial remission, NR not remission.

High intensity treatment included patients received ≥4 courses of MDAC/HDAC or HSCT, and low intensity treatment included patients received standard regimens from induction or <4 courses of MDAC/HDAC in consolidation.

### 3.3. Analyse of published gene expression microarrays validates RQ-PCR results of PDCD7 on OS survival

**Figure 5:**
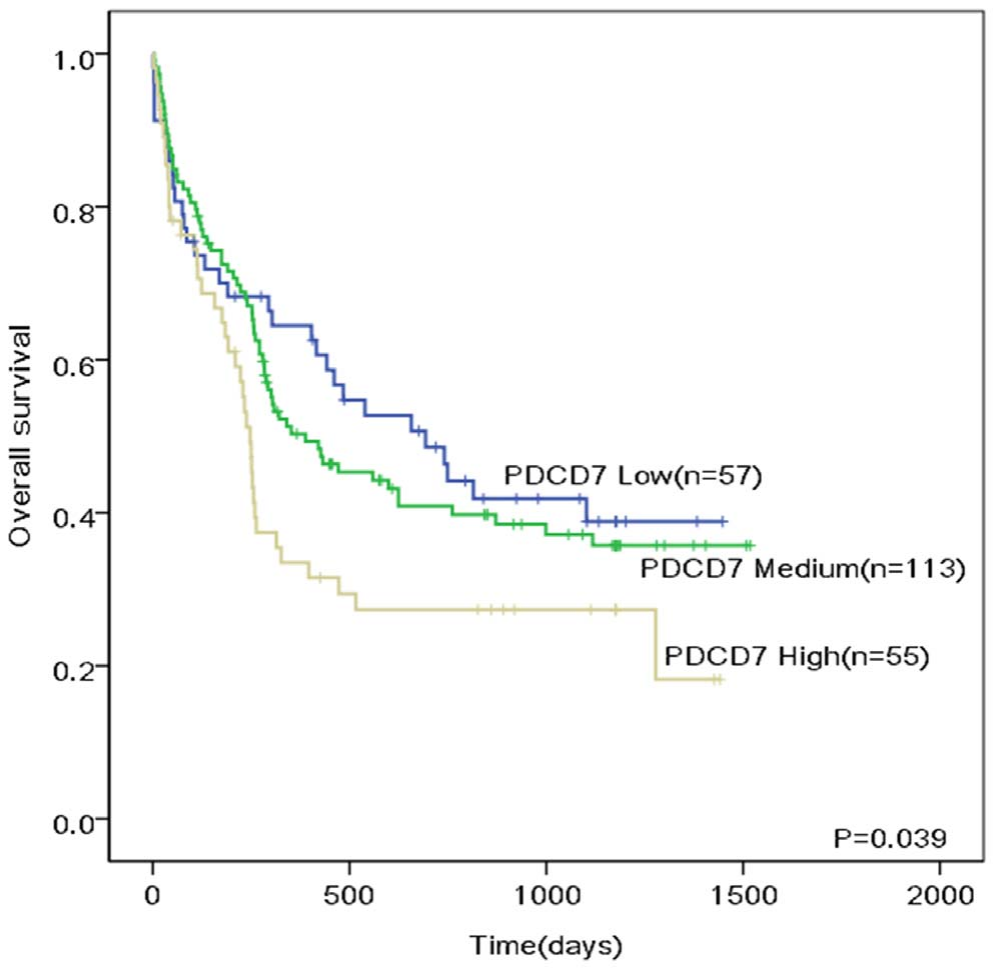
Overall survival of patients with CN-AML according to different PDCD7 expression levels from published microarray data.

For further validate that our RQ-PCR results of PDCD7 on OS estimation were generalizable to AML patients irrespective of different chemotherapies, we analysed PDCD7 expression data from 225 CN-AML patients treated according to the German AMLCG-1999 protocol, and confirmed the prediction (HR, 1.291; 95% CI, 1.013–1.645; P = 0.039; Fig. 5).

## Discussion

Prognostic evaluation system for AML has been established and improved in the past decades, some of the risk factors are already widely accepted, such as age [12], secondary AML, cytogenetic abnormalities [13], [14] and increasingly counted molecular aberrations [15]. However, accurate risk-stratification of AML at diagnosis remains difficult. For further improving the efficacy of evaluation and prediction for AML, we investigate the expression and clinical relevance of eight novel molecular markers based on our ealier study [2] and angiopoietin family member Ang2 in AML.

PDCD7 (programmed cell death 7) encoding a 59 kDa protein, mainly works in U12-type minor spliceosome to interfere the splicing procession of mRNA precursors [16]–[18]. Human PDCD7 was seldom reported concerning any specific disease, its homologue ES18 from mus musculus responded selectively to apoptosis inducing reagents [19]. Our previous gene-chip study of 6 AML patients has revealed that PDCD7 expression differs in patients with different prognosis. In our present study, AML patients had higher PDCD7 compared to non-malignant controls, and PDCD7 was positively correlated to bone marrow blast counts, which indicating a correlation between tumor burden and PDCD7 expression in AML. Moreover, high PDCD7 expression was associated with a shorter OS and RFS in CN-AML, but the wide variety of induction and consolidation therapies made it difficult to conclude the predicting efficacy of PDCD7 on response and survival in AML. In that regard, we included remission status of two inductions and treatment intensity of consolidation in multivariate analysis, and PDCD7 remained an independent risk factor for OS in CN-AML (Table 2). Furthermore, analysis of published data from 225 CN-AML patients treated in uniform protocol also displayed PDCD7 to be associated with OS, and strongly supported our results.

FIS1 (fission 1 (mitochondrial outer membrane) homolog (S. cerevisiae)) has been well established in the etiology of neurodegenerative diseases [20]–[24], and commonly regarded as mitochondrial fission inducer and promoting cell apoptosis [25]–[27]. Recent study has established a model involving the Fis1/Bap31/procaspase-8 platform [25], [28], which helped increase the calcium load of mitochondria, and resulted in cell apoptosis eventually [25]. As to solid tumors and leukemia, FIS1 was only involved in cell signaling pathways of breast cancer [29] and prostate cancer [28], except for our antecedent studies [2], [30], [31]. In the present study, AML patients with high FIS1 expression were more likely to be classified as M0/M1 FAB subtype, and have a relatively higher CR rate. Meanwhile, FIS1 showed an association with primary refractory disease in AML, especially for CN-AML, and was also validated at protein level. Hence, we concluded that FIS1 seemed over-expressed in patients with more naïve balsts and was a risk factor for early clinical response of AML.

The angiogenesis family member Ang2 was initially regarded as vascular remodeling antagonist of Ang1 [32], [33], later on, the concerted action of Ang2 and VEGF-A was noticed on endothelial cells [34], [35]. In the absence of VEGF-A, Ang2 led to endothelial cell apoptosis and vessel regression, and in the presence of VEGF-A, Ang2 promoted cell proliferation and migration. Previous studies ever came to completely controversial results of the role Ang2 played in survival estimation of AML [3]–[6], which might be caused by the unbalanced distribution of VEGF-A levels and stromal cells in bone marrow. Our study revealed Ang2, in consistent with previous study [5], a risk factor for early clinical response in AML, that the CR rate of patients with high Ang2 was 62.5% versus 82.9% of those with low Ang2 expression. However, unlike the published reports [3]–[6], Ang2 seemed have no influence on long-term survival in our study, this might be caused by the lack of VEGF-A data.

KLRF1 (killer cell lectin-like receptor, subfamily F, member1, KLRF1) expressed on the surface of most NK cells and a part of T cells [36]–[38], stimulating cytotoxicity and cytokine secretion, and was seldom reported involving leukemia. In our study, KLRF1 expression was significantly lower in AML patients as compared to non-malignant controls, which might be predominantly caused from the down-regulation of KLRF1 on NK cells or the decrease in NK cell numbers, companied with the reduced NK cell surveillance of tumors.

In conclusion, PDCD7 predicted shorter OS and RFS in CN-AML, Ang2 and FIS1 related to CR response in AML. Thereby, PDCD7, Ang2 and FIS1 may indicate a more aggressive form and poor prognosis of AML.
